# Contribution of Four Polymorphisms in Renin-Angiotensin-Aldosterone-Related Genes to Hypertension in a Thai Population

**DOI:** 10.1155/2019/4861081

**Published:** 2019-08-14

**Authors:** Pimphen Charoen, Jakris Eu-ahsunthornwattana, Nisakron Thongmung, Pedro A. Jose, Piyamitr Sritara, Prin Vathesatogkit, Chagriya Kitiyakara

**Affiliations:** ^1^Department of Tropical Hygiene, Faculty of Tropical Medicine, Mahidol University, Bangkok 10400, Thailand; ^2^Integrative Computational BioScience (ICBS) Center, Mahidol University, Bangkok 10400, Thailand; ^3^Department of Community Medicine, Faculty of Medicine, Ramathibodi Hospital, Bangkok 10400, Mahidol University, Thailand; ^4^Department of Medicine, Faculty of Medicine, Ramathibodi Hospital, Mahidol University, Bangkok 10400, Thailand; ^5^Research Center, Faculty of Medicine, Ramathibodi Hospital, Mahidol University, Bangkok 10400, Thailand; ^6^Division of Renal Diseases and Hypertension, The George Washington University School of Medicine and Health Sciences, Washington, DC, USA

## Abstract

**Introduction:**

The roles of genes in the renin-angiotensin-aldosterone system (RAAS) in hypertension, including angiotensin-converting enzyme (ACE), angiotensinogen (AGT), angiotensin II receptor type 1 (AGTR1), and aldosterone synthase (CYP11B2), have been widely studied across different ethnicities, but there has been no such investigation in Thai population.

**Materials and Methods:**

Using 4,150 Thais recorded in the Electricity Generating Authority of Thailand (EGAT) study, we examined the association of rs1799752, rs699, rs5186, and rs1799998 located in or near *ACE*, *AGT*, *AGTR1*, and *CYP11B2* genes in hypertension. We investigated their roles in hypertension using multivariate logistic regression and further examined their roles in blood pressure (BP) using quantile regression. Sex, age, and BMI were adjusted as potential confounders.

**Results:**

We did not observe associations between hypertension and rs1799752 (*P*=0.422), rs699 (*P*=0.36), rs5186 (*P*=0.49), and rs1799998 (*P*=0.71). No evidence of association between these SNPs and BP was found across an entire distribution. A nonlinear relationship between age and BP was observed.

**Conclusion:**

In Thai population, our study showed no evidence of association between RAAS-related genes and hypertension. While our study is the first and largest study to investigate the role of RAAS-related genes in hypertension in Thai population, restricted statistical power due to limited sample size is a limitation.

## 1. Introduction

Hypertension is highly prevalent globally. In 2005, the global burden of hypertension was estimated to rise from nearly 1 billion in the year 2000 to 1.6 billion in 2025 [1]. By 2010, the global burden of hypertension was estimated at about 1.4 billion, and this will potentially exceed 1.6 billion sooner than 2025 [2]. In Thailand, the Thai Burden of Disease study in 2009 reported hypertension as in the top 3 risk factors for disability-adjusted life-years (DALYs) in both males and females. According to the latest National Health Examination Survey conducted in 2015, one out of four Thais of 15 years of age and older had hypertension [3].

Hypertension is a complex disease caused by both genetic and environmental factors. The renin-angiotensin-aldosterone system (RAAS) is a hormone system that regulates blood pressure (BP) and fluid and electrolyte balance. It is commonly targeted for the treatment of hypertension [4, 5]. Therefore, on the basis of prior knowledge on biological functions, polymorphisms in candidate genes of the RAAS have been extensively studied, aiming to investigate the influence of RAAS genetic variability on hypertension [6–8]. While the roles of genes in the RAAS, including angiotensin-converting enzyme (ACE), angiotensinogen (AGT), angiotensin II receptor type 1 (AGTR1), and aldosterone synthase (CYP11B2) genes in hypertension, have been widely studied across different ethnicities [9–11], there have been limited investigations in Thai population.

In this study, we examined 4 polymorphisms in the RAAS selected because of their roles in the pathogenesis of hypertension. The reference SNP identification (rs) of ACE, rs1799752, an insertion/deletion (I/D) polymorphism, has been associated with many diseases. The D allele, which has increased activity, not related to increased generation of angiotensin II, is associated with increased risk of hypertension and preeclampsia, among others [11]. AGT is converted by ACE to angiotensin II, a potent vasoconstrictor; M235T (rs699) is a nonfunctional polymorphism, but 235 is in linkage equilibrium with −6A [12]. AGT haplotype 1, which contains the variants −217A, −6A, +507G, and +1164A, is associated with increased BP in humans and transgenic mice [12]. The prohypertensive effect of angiotensin II occurs by occupation of AGTR1, resulting in vasoconstriction and sodium retention [13]. Polymorphisms of AGTR1 such as rs5186 are associated with hypertension [14–17]. The ability of aldosterone to increase BP is caused not only by increasing renal sodium transport but also by increasing vascular smooth muscle contractility, among others, via mineralocorticoid and nonmineralocorticoid receptors [18]. Aldosterone synthase, which is needed to synthesize aldosterone, has a genetic polymorphism, CYP11B2 rs1799998 [18], that is associated with hypertension [19]. However, a recent meta-analysis was not able to show the association of the SNPs of ACE, AGT, and CYP11B2 genes and hypertension [20]. Because the associations between these SNPs and hypertension could be ethnic-dependent and the associations of these SNPs and hypertension have not been studied in the Thai population, we investigated the associations between these SNPs and hypertension in 6463 Thais.

## 2. Methods

### 2.1. Data and Study Design

The subjects were employees of EGAT (the Electricity Generating Authority of Thailand) who volunteered to participate in a health survey. In 1985, 3499 workers of EGAT (half of the total employees) were randomly enrolled as EGAT 1 cohort. In 1998, 2999 employees were randomly enrolled as EGAT 2 cohort. The age range of 35–54 years was selected in both EGAT 1 and EGAT 2. Both EGAT cohorts were surveyed in 1997-1998 with every 5-year follow-up. During the follow-up in 2002-2003, blood for genotyping was also drawn after a 12-hour fast. DNA was extracted from whole blood, and one SNP per gene was previously selected and genotyped using fluorescent probe melting analysis for rs1799752 (ACE), rs699 (AGT), rs5186 (AGTR1), and rs1799998 (CYP11B2). BP was measured twice after 10-minute rest in a seated position, using a validated automatic device. Individuals were classified as hypertensive when systolic BP (SBP) > 140 mmHg and diastolic BP (DBP) > 90 mmHg. More details of the EGAT study cohorts and the study protocols can be found in the study by Vathesatogkit et al. [21].

### 2.2. Inclusion and Exclusion Criteria

All samples with genotyping data were included in our analyses. All individuals were included in analyses of hypertension. Individuals taking antihypertensive medication were excluded from analyses of BP. This was done to avoid BP levels that are artificially lowered regardless of the genetic background.

### 2.3. Statistical Analyses

GAS (Genetic Association Study Power Calculator) was used to perform power calculations [22]. For inputs, the GAS requires the number of cases and controls, disease model, disease prevalence, allele frequency, estimated genotype relative risk, and target significance level after adjusting for the number of markers tested for association.

We first tested associations between rs1799752, rs699, rs5186, rs1799998, and hypertension using multivariate logistic regression. This was done using glm() function in R. Sex, age, and BMI were adjusted as potential confounders. An additive genetic model was used to assume an additive risk of disease for an additional effect allele. For example, the genotype for rs1799752 is coded as II = 0, ID = 1, and DD = 2, where D is an effect allele. A significant threshold of 0.0125 was used after accounting for Bonferroni correction with 4 independent candidate SNPs investigated in our study.

Associations between rs1799752, rs699, rs5186, rs1799998, and BP were further explored to investigate these genetic effects in more detail. Quantile regression (QR) was applied to examine these genetic effects on the entire distribution of both SBP and DBP. The quantile regression allows the change across the *i* ^th^ quantile of BP to be tested. This allows the specific threshold currently used to classify individuals under hypertension to be relaxed under this investigation. The rq() function from the quantreg package in R was used to perform quantile regression.

### 2.4. Systematic Search for Previous Evidence of Associations

Systematic search for previous evidence of association between the 4 SNPs and hypertension-related traits across populations was performed using the GRASP search engine [23] and the UK Biobank recently made publicly available [24].

The GRASP search software (v2.0.0.0) searches GWAS catalog data housed at the National Center for Biotechnology Information (NCBI). With periodic updates, the current version of GRASP includes available genetic association results from 2,082 GWAS papers, their supplements, and web-based contents. All associations with *P* < 0.05 from GWAS defined as ≥25,000 markers tested for 1 or more traits are included in the database.

UK Biobank is the largest prospective study in the UK following about 500,000 participants aged from 40 to 69 years. Recently, summary statistics of association across a wide range of phenotypes were publicly made available, including hypertension, SBP, and DBP. Logistic and linear regressions were applied in hypertension and BP, respectively, with an adjustment for sex and 10 principal components.

## 3. Results

### 3.1. Characteristics of Study Population

In this study, we focused on hypertension from the combined EGAT 1 and EGAT 2 data collected in 1997-1998. This first wave of data collection allows the largest sample size possible. Out of the total of 6498 individuals collected in 1997-1998, up to 6463 individuals were genotyped and used in our analyses. The summary of the characteristics of EGAT data is shown in Table 1.

### 3.2. Genetic Analyses

The summary of the genotype data in the EGAT study is shown in Table 2. Using a genome browser Ensembl, we compared frequencies of the SNPs of interest between Thai and other populations (Table 3). Frequencies of rs699 and rs1799998 reported in East Asian population are shown to be the same as in EGAT data, while these are different from those in South Asian, European, American, and African populations. For rs1799752 and rs5186, information from East Asian population was not reported. The data from the aggregated populations from the Exome Aggregation Consortium show that rs1799752 is rare, while it is a common SNP in Thai population. rs5186 is reported to have the same allele frequency in Thais and African Americans but much less than that in European Americans.

In the EGAT control group, all 4 polymorphisms are in the Hardy–Weinberg equilibrium which indicates no evidence of genotyping errors, i.e., P_hwe = 0.38 for rs1799752, P_hwe = 0.20 for rs699, P_hwe = 0.18 for rs5186, and P_hwe = 0.32 for rs1799998.

### 3.3. Power Calculation

GAS was used to perform power calculations. Hypertension prevalence was 23% on average in the Thai population [3]. Allele frequencies and a number of cases and controls are shown in Tables 2 and 3. Assuming that hypertension is a complex disease with many variants of small effect, the maximum genotype relative risk of 1.1 was used in this calculation [25]. Under an additive model, the power is 0.23 for rs699, 0.11 for rs5186, 0.48 for rs1799998, and 0.44 for rs1799752.

### 3.4. Association with Hypertension

We investigated the association between rs699, rs5186, rs1799998, rs1799752, and hypertension in up to 4150 individuals with 1331 cases and 2819 controls. Using multivariate logistic regression, no evidence of association was observed under an additive genetic model (Table 4).

### 3.5. Association with SBP and DBP

We further examined the role of these 4 polymorphisms in more detail in the entire distribution of BP. The summary of DBP and SBP across quantiles is shown in Table 5.

**Figure 1:**
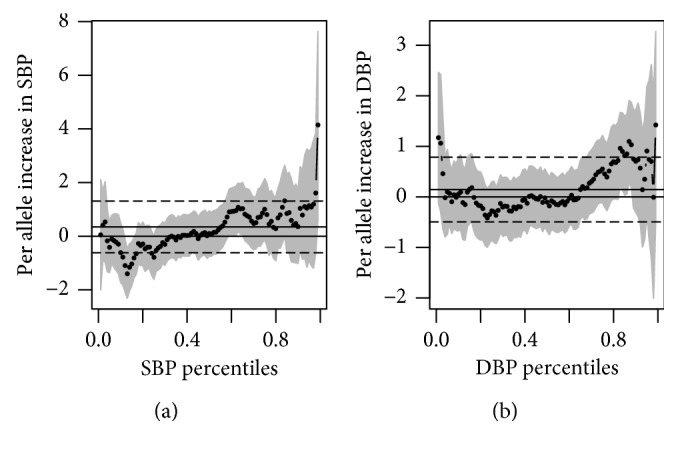
Point estimates and 95% confidence bounds (grey areas) for the increase in SBP (a) and DBP (b) per rs1799752 risk allele. The dots represent specific BP percentiles in the quantile regression model with adjustment for sex, age, and BMI. The nonzero horizontal lines represent the linear regression coefficients and their 95% confidence intervals.

ACE is a target of drugs for hypertension. However, no association was found between ACE rs1799752 and BP across an entire range using multivariate quantile regression (Figure 1). Similarly, variants of the other drug target genes, AGT (rs699), AGTR1 (rs5186), and CYP11B2 (rs1799998), were not associated with BP (Supplementary Materials (available here)).

**Figure 2:**
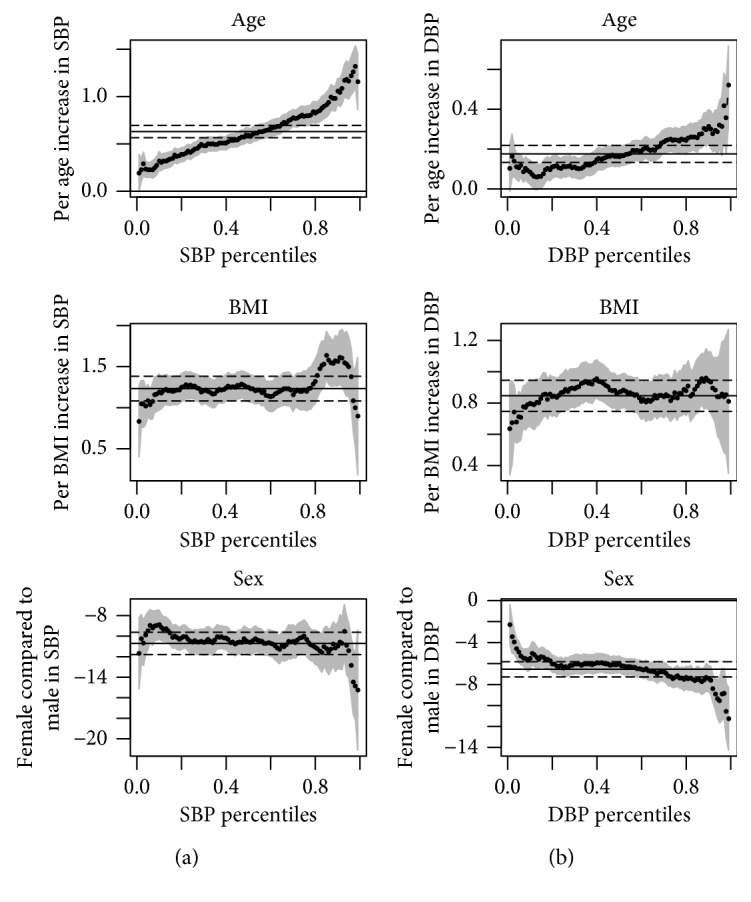
Point estimates and 95% confidence bounds (grey areas) per unit change of adjusted risk factors (sex, age, and BMI) across the *i* ^th^ quantile of SBP (a) and DBP (b) under the quantile regression when rs1799752 is included in the model.

We also studied the influence of risk factors for hypertension included in the model as potential confounders, i.e., age, BMI, and sex, on an entire distribution of BP when rs1799752 was included in the model. All risk factors were significantly associated with SBP and DBP. SBP and DBP were higher in males than those in females. We found that the influence of age on BP increased at the higher level of BP (Figure 2). This indicates possible nonlinear relations between age and each BP measurement, and a quadratic effect of age should be examined in the analyses by fitting the age-squared term. We further included the age-squared term in our model. The age-squared term was significantly associated with SBP and DBP in all models; however, this did not significantly change the results previously observed.

### 3.6. Evidence of Association in Publicly Available GWAS Studies

We systematically searched for evidence of association between our 4 SNPs of interest and hypertension-related traits across populations using the GRASP search engine [23] and a large UK Biobank recently made publicly available [24]. Unfortunately, we did not observe GWAS studies of hypertension and BP in Asian population with publicly available results. For this systematic search, we reported summary statistics from association analyses obtained mainly from Caucasian ancestry although we are aware of the difference in allele frequencies across populations, as previously shown in Table 3.

Using the GRASP search (v2.0.0.0) on GWAS catalog data, we found that only rs699 was associated with hypertension at the GWAS significant threshold (*P* < 5 × 10^−8^), with the sample size of 84,467 individuals of European ancestry [26].

We further used recently made available UK Biobank results which followed about 500,000 participants from 40 to 69 years of age. Self-reported hypertension was recorded in 27% of the whole UK Biobank population. Three out of our 4 SNPs of interest are available in UK Biobank. rs799752, previously shown as a rare SNP, was neither available in UK Biobank nor in an rAggr web-based application (http://raggr.usc.edu/) to search for its proxy SNPs. rs699 and rs1799998 were associated with SBP and DBP but not with hypertension (Table 6). By removing age and BMI from our previous models, we further reported effect sizes and effect directions from the EGAT data under the same models as in UK Biobank without an adjustment for principal components, which cannot be calculated under the limited SNPs. However, because of nonsignificant results, a comparison cannot be made.

## 4. Discussion

This study aimed to investigate the association between 4 polymorphisms in RAAS genes (i.e., rs1799752 (*ACE*), rs699 (*AGT*), rs5186 (*AGTR1*), and rs1799998 (*CYP11B2*)) and hypertension in a Thai population. In addition, their roles in SBP and DBP levels were investigated. However, we did not observe any evidence of associations between these polymorphisms and hypertension, SBP, or DBP.

The same allele frequencies of rs699 and rs1799998 were observed between Thai and East Asian populations (Table 3). rs1799752 is not in the 1000 Genomes and HapMap projects, that may be related to its low allele frequency in Caucasians; that is, a frequency less than 0.01 was reported only in the Exome Aggregation Consortium [27]. While rs1799752 in the ACE gene is rare in Caucasians, we observed a high frequency of deletion (MAF = 0.32) at rs1799752 in our Thai population.

ACE has a wide range of insertion/deletion regions [28]. The polymorphism of the ACE gene is known for the presence or absence of a 287 bp element on intron 16 on chromosome 17. Hypertension-related traits and genetic mechanisms may vary across races and ethnicities [29]. In contrast to Caucasian populations, there are a limited number of studies in Asian populations on the relationship of the ACE polymorphisms and hypertension. While there are associations between ACE I/D and hypertension in some Chinese [30, 31] and Indian [32, 33] populations, lack of such associations has also been reported [34, 35]. Other genes in the RAAS including AGT, AGTR1, and CYP11B2 have also been widely studied [36]. However, there has been no investigation on the association of polymorphisms in the RAAS and hypertension in a Thai population. In our present study on a Thai population, the association of polymorphisms in the RAAS (rs1799752 (ACE), rs699 (AGT), rs5186 (AGTR1), and rs1799998 (CYP11B2)) and hypertension was not observed.

The motivation for using QR is that it assesses how conditional quantiles of BP vary with respect to measured covariates. There is no theoretical reason to assume that the effect of the covariates is the same at different quantiles of the distribution. In our case, we observed a quadratic effect of age on BP. Because QR considers the entire conditional distribution of the dependent variable and not only its mean as in linear regression, it could provide a more complete picture of the conditional distribution than a single estimate of the center. QR also avoids the need to decide an arbitrary threshold to define the “extremes” [37]; that is, the cutoff of 140 mmHg SBP and 90 mmHg DBP of hypertensive cases can be relaxed. However, in our study, QR did not reveal an evidence of association at any particular quantile, and large confidence intervals across the range were observed.

In 2017, Ji et al. also reported a systematic search on the GWAS catalog for association between a number of polymorphisms in the RAAS and hypertension at the significant threshold level of 5 × 10^−8^. Many polymorphisms did not show an evidence of association across studies, while other polymorphisms associated with traits that have no direct connection with hypertension [38]. Nevertheless, analysis of a very large UK Biobank that was recently made available revealed an association between polymorphisms in RAAS genes and hypertension-related traits at the significant threshold level of 5 × 10^−8^, e.g., rs699 in AGT with both SBP and DBP and rs4308 in ACE with DBP [39]. This could imply that a very large sample size might be required to have an adequate statistical power to detect association in hypertension-related traits, such as shown in AGT and ACE genes.

In conclusion, we did not observe any association between 4 polymorphisms in the RAAS and hypertension in a Thai population. An effect on SBP and DBP in the entire distribution was also not found. While our study is the first and largest study to investigate the role of different polymorphisms in RAAS-related genes in hypertension in a Thai population, the sample size still restricted the statistical power. Our study suggests that either there is no association between these 4 polymorphisms in RAAS-related genes and hypertension or a much larger sample size is required to detect if there is a true association. In our study design, at least 3500 cases and 3500 controls are required to obtain an 80% power to detect if there is a true association between rs1799752 in ACE and hypertension. In addition, denser polymorphisms across these 4 genes are needed to provide a better coverage in the regions of interest.

## Figures and Tables

**Table 1:** Characteristics of EGAT data.

	Male	Female
*N*	4891	1572
Age	48.34	47.10
BMI	24.33	24.01
Hypertension	39.13%	21.18%
BP medication	10.39%	7.63%

**Table 2:** Selected SNPs in the RAAS-related genes in EGAT data.

	Chr	Location	Gene	Effect allele	Noneffect allele	*N* of genotyped samples	*N* of genotyped samples with hypertension
rs699	1	230710048	AGT	A	G	3572	1112
rs5186	3	148742201	AGTR1	C	A	4108	1305
rs1799998	8	142918184	CYP11B2	G	A	4150	1331
rs1799752	17	63488529	ACE	D (deletion)	I (insertion)	3674	1171

**Table 3:** Effect allele frequencies of selected SNPs across different populations.

	Effect allele frequency								
	Genome browser Ensembl								
	Thais (EGAT study)	East Asian	South Asian	European	American	African	African American	European American	Aggregated populations
rs699^*∗*^	0.15	0.15	0.36	0.59	0.37	0.10	—	—	—
rs5186^*∗∗*^	0.06	—	—	—	—	—	0.06	0.29	—
rs1799998^*∗∗∗*^	0.3	0.29	0.45	0.49	—	0.18	—	—	—
rs1799752^*∗∗∗∗*^	0.32	—	—	—	—	—	—	—	<0.01

^*∗*^1000 Genomes Project Phase 3: East Asian (EAS: CDX, CHB, CHS, JPT, KHV), South Asian (SAS: BEB, GIH, ITU, PJL, STU), European (EUR: CEU, FIN, GBR, IBS, TSI), American (AMR: CLM, MXL, PEL, PUR), and African (AFR: ACB, ASW, ESN, GWD, LWK, MSL, YRI). ^*∗∗*^NHLBI Exome Sequencing Project allele frequencies. ^*∗∗∗*^HapMap Project: East Asian (EAS: CHB, JPT), South Asian (SAS: GIH), European (EUR: TSI, CEU), and African (AFR: ASW, LWK, YRI). ^*∗∗∗∗*^Aggregated populations from the Exome Aggregation Consortium (ExAC).

**Table 4:** Odds ratios of developing hypertension among samples with an effect allele, as compared to samples with a noneffect allele, including their *P* values.

	Additive model	
	OR (95% CI)	*P* value
rs1799752	1.03 (0.92, 1.17)	0.60
rs699	0.91 (0.78, 1.06)	0.24
rs5186	1.07 (0.87, 1.32)	0.51
rs1799998	0.98 (0.87, 1.09)	0.67

**Table 5:** SBP and DBP across quantiles.

	The *i* ^th^ quantile				
	0	25	50	75	100
SBP (mmHg)	76.33	113.50	124.50	137.54	236.50
DBP (mmHg)	44.00	69.00	76.50	84.50	149.00

**Table 6:** Summary statistics from UK Biobank and EGAT data.

	Hypertension				SBP				DBP			
	EGAT study		UK Biobank		EGAT study		UK Biobank		EGAT study		UK Biobank	
	*β*	*P*	*β*	*P*	*β*	*P*	*β*	*P*	*β*	*P*	*β*	*P*
rs699	0.11	0.16	−5.72 × 10^−5^	0.55	0.76	0.23	−0.02	7.28 × 10^−11^	0.04	0.93	−0.02	4.15 × 10^−10^
rs5186	−0.23	0.05	5.31 × 10^−5^	0.60	−0.28	0.75	2.89 × 10^−3^	0.28	−0.71	0.20	1.25 × 10^−3^	0.64
rs1799998	−0.08	0.18	3.79 × 10^−5^	0.69	−0.27	0.57	−9.61 × 10^−3^	1.03 × 10^−4^	−0.25	0.39	−0.01	1.30 × 10^−6^

## Data Availability

The data used to support the findings of this study are available from the corresponding author upon request.

## Supplementary Materials

Supplementary Materials Supplementary Figure 1: point estimates and 95% confidence bounds (grey areas) for the increase in SBP (left) and DBP (right) per rs699 risk allele. The dots represent specific BP percentiles in the quantile regression model with adjustment for sex, age, and BMI. The nonzero horizontal lines represent the linear regression coefficients and their 95% confidence intervals. Supplementary Figure 2: point estimates and 95% confidence bounds (grey areas) for the increase in SBP (left) and DBP (right) per rs5186 risk allele. The dots represent specific BP percentiles in the quantile regression model with adjustment for sex, age, and BMI. The nonzero horizontal lines represent the linear regression coefficients and their 95% confidence intervals. Supplementary Figure 3: point estimates and 95% confidence bounds (grey areas) for the increase in SBP (left) and DBP (right) per rs1799998 risk allele. The dots represent specific BP percentiles in the quantile regression model with adjustment for sex, age, and BMI. The nonzero horizontal lines represent the linear regression coefficients and their 95% confidence intervals.
