# Supplementary material for: Contribution of Four Polymorphisms in Renin-Angiotensin-Aldosterone-Related Genes to Hypertension in a Thai Population
Source: Int J Hypertens. 2019 Aug 14;2019:4861081. doi: 10.1155/2019/4861081 (PMC6710803; doi:10.1155/2019/4861081)
Supplement: Supplementary Materials — Supplementary Figure 1: point estimates and 95% confidence bounds (grey areas) for the increase in SBP (left) and DBP (right) per rs699 risk allele. The dots represent specific BP percentiles in the quantile regression model with adjustment for sex, age, and BMI. The nonzero horizontal lines represent the linear regression coefficients and their 95% confidence intervals. Supplementary Figure 2: point estimates and 95% confidence bounds (grey areas) for the increase in SBP (left) and DBP (right) per rs5186 risk allele. The dots represent specific BP percentiles in the quantile regression model with adjustment for sex, age, and BMI. The nonzero horizontal lines represent the linear regression coefficients and their 95% confidence intervals. Supplementary Figure 3: point estimates and 95% confidence bounds (grey areas) for the increase in SBP (left) and DBP (right) per rs1799998 risk allele. The dots represent specific BP percentiles in the quantile regression model with adjustment for sex, age, and BMI. The nonzero horizontal lines represent the linear regression coefficients and their 95% confidence intervals. [file 4861081.f1.pdf]

Supplementary Figures

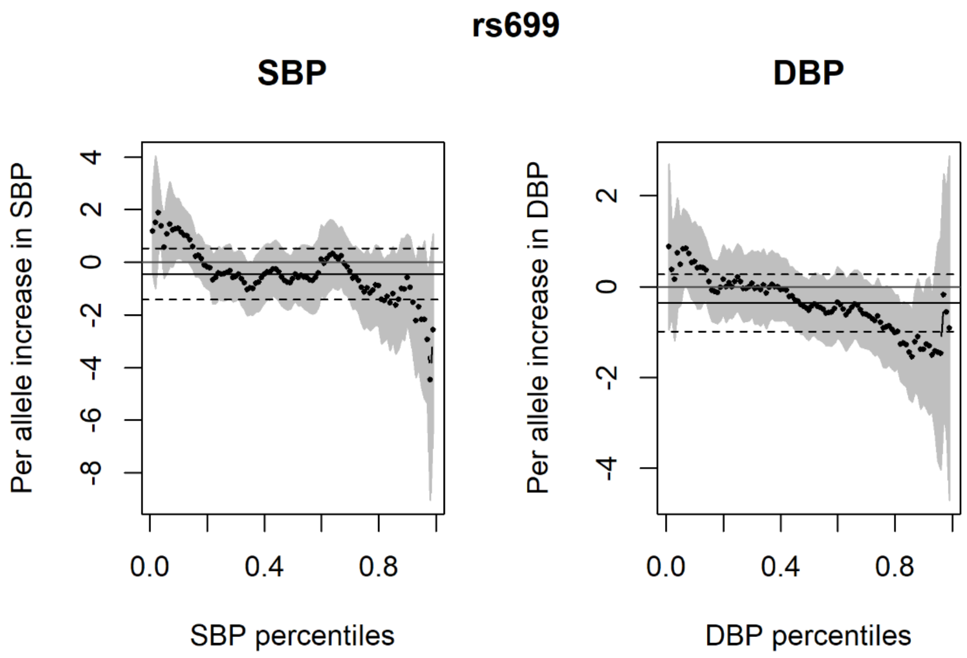

Supplementary figure 1. Point estimates and 95% confidence bounds (grey areas) for increase in SBP (left) and DBP (right) per rs699 risk allele. The dots represent specific BP percentiles in the quantile regression model with adjustment for sex, age and BMI. The non-zero horizontal lines represent the linear regression coefficients and their 95% confidence intervals.

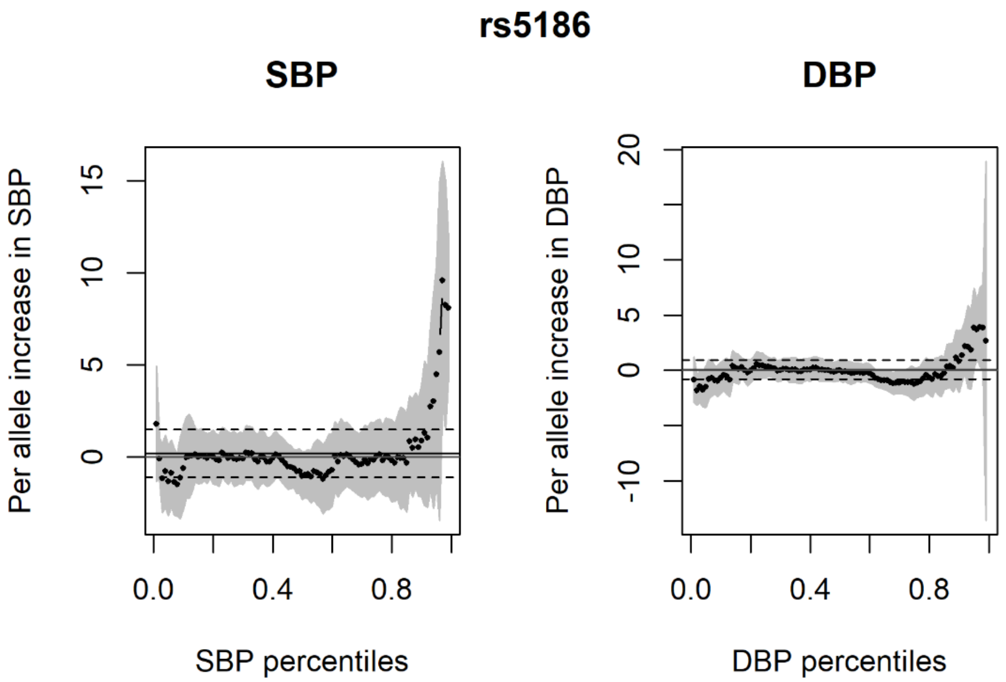

Supplementary figure 2. Point estimates and 95% confidence bounds (grey areas) for increase in SBP (left) and DBP (right) per rs5186 risk allele. The dots represent specific BP percentiles in the quantile regression model with adjustment for sex, age and BMI. The non-zero horizontal lines represent the linear regression coefficients and their 95% confidence intervals.

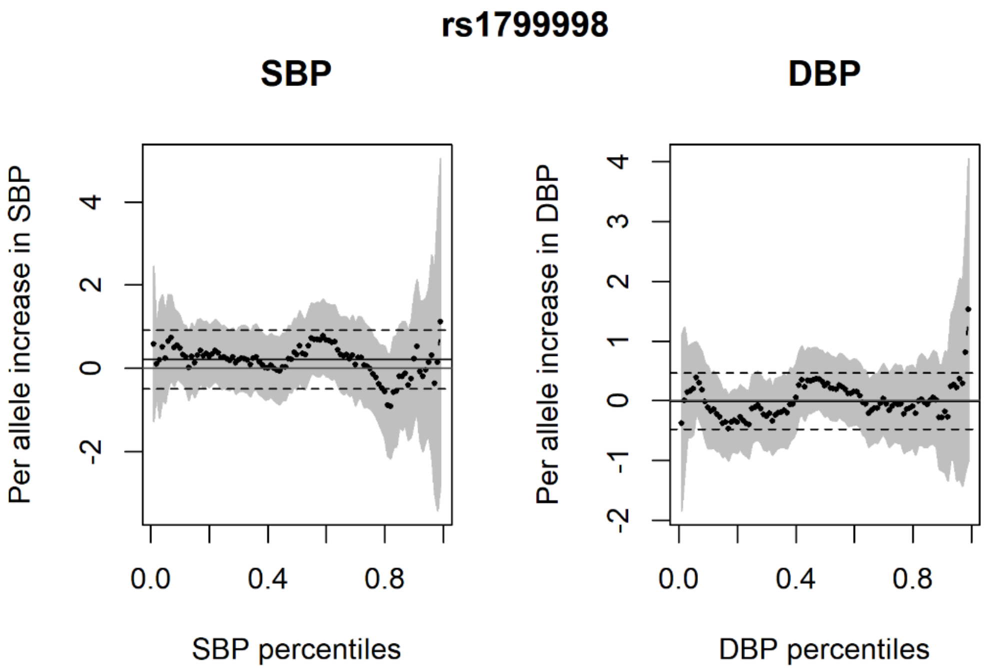

Supplementary figure 3. Point estimates and 95% confidence bounds (grey areas) for increase in SBP (left) and DBP (right) per rs1799998 risk allele. The dots represent specific BP percentiles in the quantile regression model with adjustment for sex, age and BMI. The non-zero horizontal lines represent the linear regression coefficients and their 95% confidence intervals.
